# The New York College of Dentistry

**Published:** 1870-02

**Authors:** 


*The New York College of Dentistry.*—We are gratified to learn that this institution is again in operation. The names of but two of the original members appear among the present Faculty, Drs. Abbot and Weisse. Among the new members are two of the graduates of the "Baltimore College of Dental Surgery," Dr. C. A. Woodward, Professor of Mechanical Dentistry, and Dr. James B. Littig, Demonstrator of Mechanical Dentistry. The new Faculty have our best wishes for a long and successful career.

